# Investigation of boron nanosized particles prepared with various surfactants and chitosan in terms of physical stability and cell viability

**DOI:** 10.55730/1300-0527.3449

**Published:** 2022-07-03

**Authors:** Emrah ÖZAKAR, Mehmet Semih BİNGÖL, Rukiye SEVİNÇ ÖZAKAR

**Affiliations:** 1 Department of Pharmaceutical Technology, Faculty of Pharmacy, Atatürk University, Erzurum, Turkey; 2 Eastern Anatolia High Technology Application and Research Center (DAYTAM), Atatürk University, Erzurum, Turkey

**Keywords:** Boron nitride, chitosan, nanosuspension, physical stability, cytotoxicity

## Abstract

Nanosuspensions (NS) are one of the new generation drug carrier forms developed to overcome the deficiencies of drugs with poor water solubility or insolubility and are considered to be one of the most successful approaches to formulate compounds in recent years. Boron nitride (BN) is insoluble in water and chemically more stable than carbon, it offers better biological superiority although the application of carbon structures in the biomedical field has increased in recent years. Chitosan is a polymer with excellent processability and biocompatibility thanks to its high dielectric constant. In addition, chitosan has a high affinity for metal ions. This study aims to combine BN and chitosan, which have unique properties, using six different surfactants, and to investigate their long-term stability for the use of both in medicine. In this direction, 24 different BN NS formulations were prepared. The 6th and 12th months’ stability of these formulations were studied at +25 °C, 60% relative humidity, and +4 °C. Also, the prepared formulations were evaluated by cell viability test and examined in terms of toxicity. FTIR spectra of the formulations were taken and their morphologies were characterized by SEM. Prepared NSs with Poloxamer 407 + Tween (N1 − N6) were found to be the most stable formulations for 6 and 12 months both at +4 °C and +25 °C. The fact that BN has a negative zeta potential and chitosan has a high positive zeta potential in formulations is very important in terms of their potential antimicrobial activities. The low cellular toxicity of BN NSs, especially chitosan-coated BN NSs, at higher concentrations shows that they have enormous potential in the diagnosis and treatment of diseases with boron-based compounds in the future.

## 1. Introduction

Nanotechnology involves the engineering of nanoparticles with two different approaches, the “top-down” and the “bottom-up” approach. The top-down approach essentially involves producing nanoparticles from bulk material using advanced techniques to obtain nanoparticles such as engineering and lithography. The bottom-up approach involves the formation of nanoparticles by combining atoms or molecules with various physical or chemical methods [1]. Many active substances exhibit low solubility and permeability. This situation results in low absorption and bioavailability. It is necessary to develop various new approaches for drugs with low water solubility to overcome these problems (e.g., nanosuspensions, nanoemulsions, nanoparticles, polymer micelles, liposomes, etc.) [2]. Nanosuspensions (NS) are one of the new generation drug carrier dosage forms developed to overcome the deficiencies of poorly water-soluble drugs [3]. In recent years, nanosuspensions are considered to be one of the most successful approaches to formulate compounds that are insoluble or poorly water-soluble [4] NS are unique colloidal dispersions of pure drug particles stabilized using a suitable surfactant and/or polymer [5]. NS offers a larger surface area that allows drugs to increase their rate of dissolution, solubility, and bioavailability [6].

Boron nitride (BN) is chemically more stable than carbon, it offers better biological superiority although the application of carbon structures in the biomedical field has increased in recent years [7], the use of BN has not yet been fully explored. The most important reason for this is that BNs for biological applications do not dissolve sufficiently in aqueous environments due to their high chemical stability [8]. This solubility problem is eliminated by formulations such as nanosuspension designed with nanotechnology. Studies of the cytocompatibility of BNs with human neuroblastoma cells show that they have no adverse effects on cell line viability, metabolism, and replication. In addition, fluorescent labeling of BNs with quantum dots and monitoring their cellular uptake are among the carried studies [9,10]. All these show that nanostructured BNs can be applied in cellular therapy, drug delivery, gene therapy, and other biomedical and clinical applications [11].

One of the important goals of modern nanomedicine is the controlled release and targeting of drugs. These can be easily accomplished by physical and chemical modification of BNs. Thanks to the magnetic properties of BNs, drugs can be targeted and systems can be designed that can be physically directed to the target area and carry the accompanying drugs. The unique properties of BNs, along with chemically or physically coatings, can be useful in therapeutic targeting. For example, the boron in the boron neutron capture therapy which is a cancer treatment is effectively used in the treatment of various cancer types, including glioblastoma, multiforme, and melanomas [12, 13]. Moreover, the piezoelectric properties of BNs make them an attractive candidate to be exploited as a bionanotransducer for stimulating cells [14]. Clearly, extensive in vivo and preclinical testing is needed to investigate the biomedical use of BNs. However, the applications of these nanostructures in nanomedicine have enormous potential in the treatment and diagnosis of various diseases [15].

Chitosan is a straight-chain natural copolymer structurally composed of D-glucosamine and N-acetylated-glucosamine obtained by partial deacetylation of chitin [16,17]. Scientific studies on chitosan have increased since 1990. Their applicability in different areas of use has attracted attention [18]. It has found very important areas of use in wastewater treatment [19], cosmetics [20], textile industry [21], agriculture [22], food packaging, and medicine [23]. Chitosan has the ability to increase viscosity, increase solubility in various media, polyoxy salt formation, polyelectrolytic behavior, film and chelate formation, and unique optical properties. Chitosan is a polymer with excellent processability and biocompatibility thanks to its high dielectric constant. In addition, chitosan has a high affinity for metal ions. Thanks to this feature, it has been used to prepare metal nanocomposites in various fields in electronics, biomedicine, optical devices, and as a catalyst. In the past, the extraordinary properties of each individual component have been observed synergistically in chitosan-prepared materials of polymers, oxidizing agents, and metal nanoparticles [16]. In addition, the applicability of chitosan in many different biomedical fields, from the proliferation of the gums to coronary stent applications, from its antibacterial effects to the regeneration of endothelial cells, has been proven by recent studies [24]. As chitosan has broad-spectrum antibacterial activity and strong biocompatibility, functionalized metallic and polymeric nanoparticles can be designed with chitosan. In this way, the biological activity of nanoparticles can be increased. Due to its cationic structure, chitosan has the ability to damage bacterial biofilms by penetrating the negatively charged bacterial membrane. Thus, as a result of surface modification of functionalized nanoparticles with chitosan molecules, the antimicrobial activities and biocompatibility of nanoparticular systems can be greatly increased [25].

This study aims to combine BN and chitosan, which have unique properties, using 6 different surfactants, and to investigate their long-term stability for the use of both in medicine. In this direction, 24 different BN NS formulations were prepared. The stability of nanosuspensions depends on the hydrophobicity of the drug and the stabilizer. A similar hydrophobicity results in better surface coverage and thus better stabilization. In this study, the effects of stabilizers and chitosan on the stabilization of BN NSs were investigated. For this reason, hydrophilic (such as SLS, Poloxamer 407, and Tween 20) and moderately hydrophobic (such as PVA and Tween 60) stabilizers were selected and studied in the preparation of NSs [26]. The 6th and 12th months’ stability of these formulations at +25 °C, 60% relative humidity, and +4 °C were compared with freshly prepared samples in terms of zeta potential, particle size, and polydispersity index (PDI) values. Also, the prepared formulations were evaluated by cell viability test and examined in terms of toxicity. FTIR spectra of the formulations were taken and their morphologies were characterized by SEM. All obtained experimental data from the formulations were statistically analyzed. The effects of particle size and zeta potential on the stability of the formulations and the causative factors were evaluated in detail.

## 2. Materials and methods

### 2.1. Materials

Boron nitride, medium molecular weight (MW) chitosan, Tween 20, acetic acid, Poloxamer 407, sodium lauryl sulphate (SLS), polyvinyl alcohol (PVA, high MW), and dimethyl sulphoxide (DMSO) used in the preparation of nanosuspensions were purchased from Sigma-Aldrich (USA). Tween 60 and PVA (low MW) were purchased from Merck (Germany). Gibco™ DMEM/F12 and Gibco™ FBS used for cell culture studies were purchased from ThermoFisher Scientific (USA). Penicillin/Streptomycin, PBS, Trypsin/EDTA, MTT Cell Growth Assay Kit CT-02, and Triton™ X-100 were purchased from Sigma-Aldrich (USA). Deionized water (Direct-Q^®^ 3 UV Millipore, Merck) was used in all formulations (18.2 MΩ·cm, TOC ≤ 4 ppb).

### 2.2. Development of BN NS and chitosan-coated BN NS formulations

Firstly, BN was dissolved with a minimum amount of DMSO in a glass vial. Tween 20 or Tween 60 has been added to it in varying proportions. It was homogenized by vortexing (IKA^®^, MS2 Minishaker, Germany). Afterwards, ultrasonication (Sonorex RK 100 H, Bandelin, Germany) was applied in an ice bath for 5 min. After this process, the BN mixture obtained was added dropwise with the help of a 22 G injector into purified water containing different amounts of SLS, Poloxamer 407, low MW PVA, or high MW PVA under the ultrasonic probe (Sonopuls HD 2070, Bandelin, Germany). In this process, NSs were obtained by the nanoprecipitation technique [27,28]. The obtained NSs were centrifuged at 13,000 rpm for 45 min at 15 °C and then washed at least three times with distilled water to remove excess DMSO and surfactant residue. The NSs that resulted were frozen at −20 °C overnight. Finally, it was lyophilized (Martin Christ, Alpha 1–4 LD plus, Germany) for 24 h and stored in dry powder form in a dark environment that was moisture-proof for use in experiments. During the preparation of chitosan-coated BN NSs, BN was dissolved with a minimum amount of DMSO in a glass vial. Tween 20 or Tween 60 has been added to it in varying proportions. In a separate vial, chitosan was dissolved with 1 mL of acetic acid (1%, w/v) and added to the mixture containing BN. The resulting mixture was homogenized by vortexing for 5 min. The following procedures were carried out as described above. Experiments were run with at least three repetitions. The formulations and their components are shown in Table 1.

### 2.3. Yields of NS formulations

The amounts of all lyophilized NS formulations were evaluated and their yields were calculated by proportioning the amount of substance added in the initial step. This process was carried out in three repetitions for each NS formulation [27].

### 2.4. Zeta potential, PDI, and particle size analysis of NS formulations

Zeta potential is an important factor influencing the long-term stability of colloid dispersion systems [2]. All freshly prepared formulations of BN NSs and chitosan-coated BN NSs were diluted with pure water and analyzed for particle size, zeta potential, and PDI using Zetasizer (Malvern, Zetasizer, Nano ZS, UK). Zeta potential was estimated on the basis of electrophoretic mobility under an electrical field. All measurements were performed in triplicates at room temperature.

### 2.5. Morphologies of NS formulations

SEM (Zeiss, Sigma 300, Germany) analyzes were performed to examine the morphological structures of lyophilized BN and chitosan-coated BN NSs. For this purpose, lyophilized samples of BN, chitosan, BN NS, and chitosan-coated BN NSs were coated with 100 Å thick gold and examined. Structural similarities and differences between pure substances and formulations were determined.

### 2.6. FTIR analysis of NS formulations

This analysis was carried out in order to determine that there is no chemical interaction between the components that make up the formulations and BN. FTIR spectra of pure BN and prepared BN NS and chitosan-coated BN NSs were obtained directly from lyophilized powder samples with an FTIR (Bruker, VERTEX 70v, USA) spectrophotometer using attenuated total reflectance (ATR) mode. Measurements were performed in a spectral range of 400–4000 cm^−1^ with a resolution of 4 cm^−1^.

### 2.7. Evaluation of long-term stability of NS formulations

Twenty-four different formulations of BN NS and chitosan-coated BN NS, prepared in at least three replications, were evaluated for stability tests at +25 °C temperature with 60% relative humidity and +4 °C temperature conditions for 12 months [29, 30]. Each formulation group was remeasured for zeta potential, particle size, and PDI data at 6 and 12 months and compared with the results of freshly prepared and measured formulations. In this way, the effects of the excipient and chitosan were determined that were used in the finding of the most stable formulation.

### 2.8. Cell culture

Cell culture experiments with human neuroblastoma cells (SH-SY5Y, ATCC CRL-2266™) were carried out at Erzurum Technical University High Technology Application and Research Center (YUTAM). SH-SY5Y cells were cultured in a DMEM/F12 medium containing 10% FBS and 1% Penicillin/Streptomycin. The cell culture was incubated in a carbon dioxide incubator (CelCulture^®^, CCL-170B-9, ESCO, Singapore) at 37 °C and 5% CO_2_ conditions. SH-SY5Y cells were harvested with Trypsin/EDTA and seeded in a 96-well seeding plate at approximately 1 × 10^4^ cells per well. The cells’ medium was replaced every 2–3 days, and they were passaged at 80% confluence. All cell lines were purchased between 2020 and 2021 years, and all experiments were performed with mycoplasma-free cells, which were routinely tested using PCR analysis.

### 2.9. In vitro cytotoxicity assay

The MTT test is used to measure metabolic activity at the cellular level as an indicator of cell viability. MTT (3-(4,5-dimethylthiazol-2-yl)-2,5-diphenyltetrazolium bromide) is a yellow tetrazolium salt. Living cells contain enzymes that reduce MTT to formazan crystals. The darkness of the color in the purple solution, which is formed by dissolving the formazan crystals with a solvent, is related to the number of living cells. Cell viability is quantified by measuring the absorbance of this solution at 570 nm. For this purpose, 500, 250, 125, 62, 31, 16, 8, 4, 2, 1 μg/mL samples taken from the selected formulations (N1, N2, and N3, Table 2) were applied to all wells in triplicate with a total volume of 100 μL in each well. Positive control (PC) wells were treated with 1% Triton™ X-100. Negative control (NC) wells were grown in only medium components. At the end of the 24-h incubation period at 37 °C and 5% CO_2_ conditions, the old medium was removed from the wells in the seeding plate. Formazan crystals in each well were dissolved with 100 μL of DMSO. Each well was measured at 570 nm with the aid of a microplate reader (Epoch™, BioTek, USA). The results were evaluated according to the absorbance of the control cells. The formulations used in the cell viability test and their contents are shown in Table 2. The percentage of the viable cells was calculated using the following formula:


$$\begin{array}{l} \text{For control}=(\text{Mean OD control}/\text{Mean OD control})\times 100=100\%\\ \text{As for treatment}=(\text{Mean OD treatment}/\text{Mean OD control})\times 100=\text{Cell viability}\%\\ {\;}^{★}\text{OD}=\text{Optical density},\text{absorbance} \end{array}$$

### 2.10. Statistical evaluations

Intergroup statistical evaluations of NS formulations in terms of zeta potential, PDI, particle size, and stability were made with a One-way ANOVA test using IBM SPSS^®^ 20 program. The obtained results from cell culture studies were made with validated Dunnett’s test. All other calculations were determined as arithmetic mean (Ⴟ) and standard deviation (SD). The statistical significance level was accepted as p < 0.05.

## 3. Results and discussion

### 3.1. Development of BN NS and chitosan-coated BN NS formulations

Our study aims to design long-term stable nanoparticle formulations for known medical uses of boron nitride and chitosan and to carry out characterization and cytotoxicity studies on the most stable ones among these formulations. Therefore, this study was carried out in two steps. Firstly, formulations were developed through preformulation studies (Table 1) and subjected to long-term stability testing (12 months). At the end of these studies, the three most stable formulations were determined, and then characterization and cytotoxicity studies were performed with these formulations. Here, the characterization properties of these three formulations (N1, N2, and N3) were given in detail, and the cytotoxicity study was carried out to get a preliminary idea for possible future medical applications.

**Figure 1:**
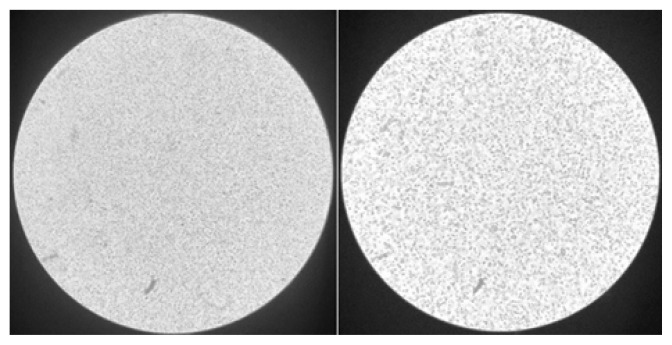
BN NSs (N4, left) and chitosan-coated BN NSs (N5, right) optical microscope images (100x).

The top-down technique was applied to prepare BN NS and chitosan-coated BN NS formulations. DMSO was chosen as a water-miscible organic solvent to easily dissolve BN and obtain smaller particles in nanoprecipitation [26]. After all formulations (N1–N24) were prepared as indicated in Table 1, their optical images were examined to observe their structures and have an idea about particle sizes (Figure 1). One for each group, BN NS, and chitosan-coated BN NS were taken images with an optical microscope (Zeiss, Primo Star, Germany) at 100× magnification power. The size of the BN NS in the images was found to be compatible with the Zetasizer results. Likewise, chitosan-coated BN NSs were observed to increase in size with the effect of the coating. Similarly, in the literature, an increase is observed in the size of NS formulations prepared by coating with chitosan [10,31].

There is a positive correlation between the molecular weight of chitosan and the particle size. It has been observed that the high MW of this polymer resulted in the formation of larger particles in studies. In our study, the medium MW form of chitosan was preferred for this reason [32]. Gan et al. showed that the particle size increased in the following order low MW chitosan < medium MW chitosan < high MW chitosan, which was independent of chitosan concentration. On the other hand, the correlation between molecular weight and zeta potential is inversely related to chitosan. In other words, as the molecular weight increases, the zeta potential values decrease [33]. However, some recent findings suggest that other factors may affect it. It cannot be assumed to be a linear relationship in all cases [34]. Hu et al. reported no significant change in particle sizes when chitosan with molecular weights of 50, 100, and 150 kDa was used. However, it was observed that the particle size decreased as the chitosan molecular weight increased from 30 to 50 kDa. However, it was reported that the particle size increased while the chitosan molecular weight increased from 150 kDa to 300 kDa, too [35].

### 3.2. Yields of NS formulations

The yields of all NS formulations are shown in Table 3. As a result of these data, it is seen that BN NSs and chitosan-coated BN NSs are prepared with high efficiency. All formulations were examined and it was seen that both BN NSs and chitosan-coated (10 mg) BN NSs were prepared with a high yield of over 80%. However, the decrease in yields of 20 mg chitosan-coated BN NSs may be due to the presence of excess chitosan in the supernatant after centrifuging the NSs.

### 3.3. Zeta potential, PDI, and particle size analysis of NS formulations

High zeta potential values must be achieved to provide a high energy barrier and achieve stability. For this purpose, zeta potential values for formulations are extremely important in terms of their stability. A higher zeta potential value achieves stabilization and tends to prevent particle aggregation. It is known that nanoparticles with a larger potential charge undergo a much higher repulsion [36]. The zeta potential, PDI, and particle size analysis results of the prepared formulations are shown in Table 3.

The zeta potential value is a very important parameter in evaluating the stability of nanosuspensions. A zeta potential value greater than ±25 mV is considered to show very good stability when nanoparticles are stabilized with low MW stabilizers mainly by electrostatic interaction. However, if stabilization is achieved with high molecular weight stabilizers (steric stabilization), good stabilization may not be achieved because sufficient repulsive forces are not formed only with zeta potential values of ±20 mV or lower [37].

The main parameter affecting the uptake of nanoparticles into the cell is their size. In general, the uptake efficiency of nanostructures decreases with increasing particle size. Particularly, particles of 200 nm or less are taken into the cells by the clathrin-mediated pathway, while larger particles are taken up into the cells via caveolae-mediated endocytosis [38,39]. However, results opposing this general idea have also been reported. For example, it has been shown that 100 nm spherical nanoparticles can be taken up via actin-dependent but clathrin-independent pathways, similarly, nanoparticles with 500 nm size and larger particles can be taken up with clathrin. These exemplary studies, among others, show that it is difficult to establish a general rule about how nanoparticle size affects cellular uptake [40]. Unlike these, while it is believed that nanoparticles larger than 200 nm cannot be taken up by nonphagocytic cells, the opposite observations are also frequently reported. For example, it has been reported that even 3 μm cubic particles can be taken up by HeLa cells. Until now, only a few studies have investigated in detail how different cell types take up nanoparticles of different sizes, but due to the small number of studies, it is difficult to make a general decision [39,40].

In our study, it was observed that the nanoparticles prepared with BN generally varied between 420–741 nm according to the surfactant type. Mainly, it was determined that the size of nanoparticles (N1 and N4) prepared using different Tweens together with Poloxamer 407 changed significantly (p < 0.05). However, in nanoparticles (N7 and N10) prepared with the use of different Tweens together with SLS, it was observed that there was no significant difference in sizes (p > 0.05). It was also determined that the size of the nanoparticles prepared using high MW PVA (N13 and N16) and nanoparticles prepared using low MW PVA (N19 and N22) decreased significantly with the hydrophilization of the Tweens (p < 0.05).

At the same time, the sizes of nanoparticles coated with chitosan were found to be between 422–1645 nm. The sizes of nanoparticles prepared using Poloxamer 407 and SLS decreased significantly depending on the increase in the amount of chitosan (10 to 20 mg, p < 0.05). It was also determined that the size of the nanoparticles prepared with PVAs (high MW PVA and low MW PVA) did not change significantly due to the increase in the amount of chitosan (p > 0.05). It was determined that the particle sizes of the chitosan-coated BN NSs prepared with PVAs were significantly smaller when compared to the nanoparticles prepared with SLS and Poloxamer 407 (p < 0.05).

Besides particle size, the net charge acting on the particle is an important parameter that can greatly affect the behavior of nanoparticles on cells [41]. In general, positively charged nanoparticles undergo cellular uptake more efficiently than neutral and negatively charged nanoparticles [42]. However, this is not a general opinion, other studies showing the opposite have also been reported [43]. In addition, it has been reported that cellular uptake increases with increasing absolute charge density [44].

In one of the nanoparticle studies with BN in the literature, the zeta potential value was found to be −5.98 ± 1.28 mV. In our studies, it was found that the zeta potential values of our formulations obtained with Poloxamer 407 and SLS were much higher and were above the threshold limit for steric stabilization [10]. In particular, it has been observed that BN NSs provide steric stabilization due to their lower value of ±30 mV, while chitosan-coated BN NSs stabilize the particles by forming an electrostatic barrier with a zeta potential higher than ±30 mV [2].

It should be noted that many studies investigating the effect of particle charge or other nanoparticle properties, such as size, on cellular interactions have been conducted in vitro. The zeta potential charge of nanoparticles can revert to their tendency to be neutral in a biological environment. It has been reported that nanoparticles, which have different charges in the aqueous medium, can have a nearly neutral or similar charge when exposed to the biological medium. Therefore, it is important to determine the behavior of nanoparticles in biological environments [39].

### 3.4. Effect of surfactants on zeta potential and particle size of NS formulations

Nanosuspensions are known to have physical instability due to their agglomeration [45]. This stability problem of nanosuspensions can be overcome by using a suitable stabilizer. Stabilizers can be nonionic polymers such as PVA, PVP, and hydroxypropyl methylcellulose (HPMC), as well as surfactants such as Poloxamer, Tween. Apart from these, ionic surfactants such as SLS can also be used [46].

A steric stabilization is achieved by adsorption on the surface of nanoparticles via water-soluble surfactants and polymers. In this way, the nanoparticles exhibit a slower Brownian motion during storage, and the particles coalesce to form aggregates which delays and prevents phase separation [47].

On the other hand, studies reported in the literature have suggested that combining surfactants and polymers in nanoformulations provides more excellent stability in the final product. They indicated that, in addition to creating a steric barrier, it would prevent aggregation better with the electrostatic repulsion effect and thus preserve the quality of the product for a more extended period of time [48]. Based on these data, we used both nonionic and ionic surfactants (such as Tween, Poloxamer 407, and SLS) and polymers (such as PVA and chitosan) to prepare BN NSs and examined their effects on the NS stability. Considering that as the amount of stabilizer increases, the zeta potential decreases, and the particle size increases, we wanted to see the effects on the stability of NSs by working with the same amount of surfactants [36].

The results in Table 3 showed that BN NSs could be successfully prepared with 6 different stabilizers and chitosan. Each formulation gave different results, especially in terms of zeta potential and particle size. It was observed that NS formulations containing only BN (N1, N4, N7, N10, N13, N16, and N19) had a negative zeta potential due to the negative charge density from BN. This negative zeta potential is greater than −20 mV with surfactants of more hydrophilic character and lower MW (such as Poloxamer 407, Tween 20, and SLS), while with surfactants of moderate hydrophilic character and higher MW it is higher than the −20 mV (as in PVA and Tween 60). Here, especially with the combination of surfactants with more hydrophilic character, stabilization was achieved through electrostatic interaction, and lower negative zeta potential was obtained (as in N1, N7, and N10) [37]. Electrostatic stabilization was achieved with the combination of a hydrophilic and a hydrophobic surfactant, resulting in a significant increase in the zeta potential and a positive shift (N4, N13, N16, N19, and N22). Considering that positively charged surfaces attract more bacteria, the formulations we have prepared can generally be used in medical treatments. In particular, they can be functionalized as antimicrobial surfaces for Gram-negative and Gram-positive strains [49].

PVA is a water-soluble synthetic polymeric stabilizer that adsorbs on nanoparticles during particle size reduction and acts as a protective colloid [50]. The decrease in zeta potentials in all BN NSs prepared using PVA may be due to the adsorption ability of PVA to the surface of the particles, the presence of acetate groups in its structure, and the shift in the shear plane. However, in chitosan-coated BN NSs prepared with PVA, there is also a positive zeta potential and a situation that creates a high electrostatic and steric barrier above the +50 mV limit. Here, too, it is seen that PVA and chitosan interact well and transform it into a structure with high zeta potential [51]. In a study, it was found that nanoparticles prepared using SLS were smaller in size and exhibited a narrower particle distribution compared to other surfactants. It has been reported that anionic surfactants (such as SLS) provide sufficient electrical charge to induce electrostatic repulsion for colloidal nanoparticles [50]. Our study determined that BN NSs prepared with SLS had the smallest particle sizes among all NS formulations (such as N7 and N10).

As can be seen in Table 3, the sizes of BN NSs were found to be between 420–741 nm on average. When we examined BN NSs in terms of particle sizes, it was observed that the particle size decreased significantly as the hydrophilicity of the surfactants included in the formulation increased. For example, the size of NSs prepared using Poloxamer 407, and Tween 20 in N1 formulation was found to be approximately 60 nm smaller than the size of NSs prepared using Poloxamer 407 and Tween 60 (N1 < N4). This situation was also seen in other NS formulations (N7 < N10, N13 < N16, N19 < N22). It was determined that the particle sizes of formulations prepared with Poloxamer 407 and Tweens (N1 and N4) increased significantly (N1 > N7, N4 > N10) compared to formulations prepared with SLS and Tweens (N7 and N10). This difference can be explained by SLS being more hydrophilic than Poloxamer. It was also determined that the particle sizes of formulations prepared with low MW PVA and Tweens (N13 and N16) were significantly reduced compared to the particle sizes of formulations prepared with high MW PVA and Tweens (N19 and N22) (N19 > N13, N22 > N16). This difference can be explained by the lesser side chain length and more hydrophilic nature in low MW PVA.

Poloxamer is the most commonly used emulsifier or solubilizer nonionic polyoxyethylene-polyoxypropylene block copolymer in pharmaceutical technology. They vary according to their molecular weights, the ratios of hydrophilic ethylene oxide and hydrophobic propylene oxide groups. Their low melting point, high surfactant properties, and safety in oral administration make them ideal for drug formulations. Their tendency to self-assemble forms micelles and increases hydrophilicity. It provides a great advantage for the solubility of slightly soluble drugs in water [52]. In our study, formulations prepared using Poloxamer 407 for water-insoluble BN were found to have a negative zeta potential (N1 and N4). Since the PDI values were below 0.3, it was determined that it exhibited a uniform size distribution. High positive zeta potentials and narrow size distributions were obtained in chitosan-coated formulations (N2, N3, and N5, N6).

### 3.5. Effect of chitosan on zeta potential and particle size of NS formulations

As can be seen in Table 3, it has been observed that the negative zeta potential arising from BN and hydrophilic surfactants used in NS formulations coated with chitosan shifts to positive with the presence of chitosan amine groups. This situation has also been reported in many studies in the literature [10, 37]. It has also been observed that this value even exceeds the +50 mV limit in nanoparticle formulations prepared with the presence of hydrophilic stabilizers. It is thought that the high zeta potential value obtained by the formulations prepared with Poloxamer 407 (N2, N3, N5, and N6) when coated with chitosan is due to the excess of hydrophilic groups in the structure and the strong electrostatic interaction with chitosan. The shift to the positive value in the zeta potential of NS formulations (N8, N9, N11, and N12) prepared with SLS was found to be significantly lower than formulations prepared with Poloxamer 407. The reason for this may be due to the competitive inclusion of sodium in the structure of the stabilizer and the amine groups in the structure of chitosan. It was determined that chitosan-coated NSs prepared with moderate hydrophilic stabilizers (such as low MW PVA and high MW PVA) showed a significantly higher zeta potential value in formulations containing Tween 20 compared to formulations prepared with Tween 60. In formulations prepared with PVA, the zeta potential of the coating with chitosan increased significantly with the increase of the molecular weight of the PVA, and this may be due to the higher stability in the electrostatic stabilization of the long polymeric chains in the PVA structure.

Table 3 shows that the average sizes of 10 mg of chitosan-coated BN NSs were between 473 – 1645 nm. Likewise, the sizes of 20 mg of chitosan-coated BN NSs were found to be between 422 and 898 nm. It was determined that there was a significant difference between the particle sizes of NSs prepared using 20 mg of chitosan and the sizes of BN NSs prepared by coating with 10 mg of chitosan. Although the amount of chitosan increased, a parallel increase in particle size was not observed. The reason for this situation is thought to be due to the fact that enough chitosan is dispersed and coated on the surface of the NSs, and there is no surface that is not coated with chitosan. Compared to NS formulations prepared with Poloxamer 407 (N2, N3, N5, N6) and SLS (N8, N9, N11, N12), the particle sizes of chitosan-coated BN NSs prepared with both PVA were found to be significantly more uniform and their size distributions narrower. This situation is similar to PDI values. There is a significant difference in particle size between the sizes of chitosan-coated BN NSs prepared using high MW PVA and Tween 20, and the sizes of chitosan-coated BN NSs prepared using low MW PVA and Tween 20. This difference may have resulted in the more hydrophobic structure in the coating with chitosan formation of NSs of high MW PVA. However, there was no significant difference between the sizes of chitosan-coated BN NSs prepared using high MW PVA and Tween 60, and the sizes of chitosan-coated BN NSs prepared using low MW PVA and Tween 60. This situation shows that the molecular weight of PVA has no effect in the presence of Tween 60. However, the sizes of chitosan-coated BN NSs using both high MW PVA and low MW PVA were found to be smaller by using Tween 20, while the particle sizes were found to increase significantly in those prepared using Tween 60. In other words, it is thought that Tween 60, which is more hydrophobic and viscous, is caused by electrostatic interactions during NS formation.

In a similar study prepared with chitosan in the literature, it was observed that the size distribution of the formulation was above 1 micron and that larger particles were formed. Given these, it is thought that this broad particle size distribution is most likely due to the formation of agglomerates [37]. In our study, it was observed that the particle size approached and even exceeded one micron in general in the coating studies of NSs with chitosan. It can be concluded that chitosan-coated NSs of 1 micron and above form aggregates due to insufficient surfactant.

The molecular weight and viscosity of chitosan affect the sizes of the NSs. In fact, particle size can be reduced by using lower MW chitosan [53]. Low MW chitosan has been reported to have a higher water solubility and, together with shorter polymer chains, contributes to the formation of smaller particles compared to high MW chitosan [54]. In our study, it is reported that the positive zeta potential of chitosan-coated BN NSs is proof of polysaccharide coating. In addition, it was observed that there were no significant changes in zeta potentials with the increasing amount of chitosan in chitosan-coated formulations. This may be due to a similar degree of deacetylation of chitosan [55].

Increasing the polymer concentration leads to a gradual increase in NS diameter while maintaining the size distribution. An increase in polymer concentration leads to an increase in viscous forces. Viscous forces resist stresses in the organic phase and directly affect the final size and size distribution of the particles. This may be the reason why the size increased, and the size distribution was constant due to the increased amount of chitosan in our study. Adding polymer to the structure (such as chitosan) increases the size and the viscosity of the organic phase, as well as the diffusion resistance against drug molecules from the organic phase to the aqueous phase. Thus, less time is left for the drug to diffuse through the polymer-coated nanoparticles. This situation reduces drug loss by diffusion and increases the drug content [56]. Considering the sizes of the NSs obtained in our study, the formulations can be used in medical treatments because the positively charged surfaces attract more bacteria surface. In particular, they can be functionalized as antimicrobial surfaces for Gram-negative and Gram-positive strains [49].

### 3.6. Morphologies of NS formulations

**Figure 2:**
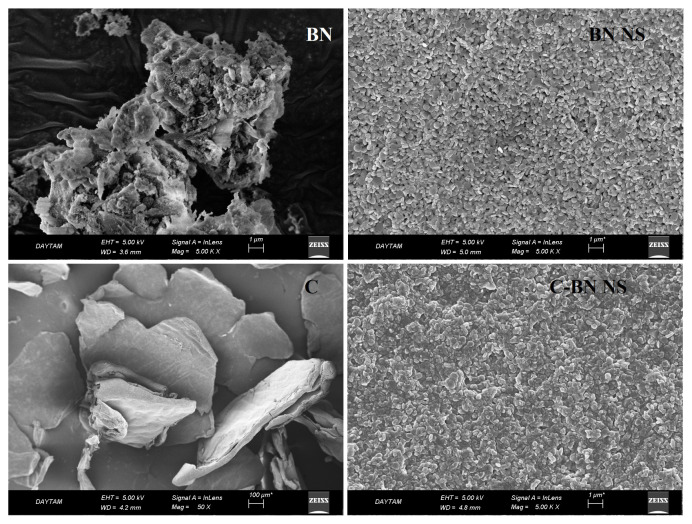
SEM images of pure substances and formulations (upper-left BN, upper-right BN NS, bottom-left chitosan (C), bottom-right chitosan-coated BN NS).

The pure BN, pure chitosan, and most stable NS formulations (N4; BN NS and N5; chitosan-coated BN NS) which were obtained as a result of stability studies were investigated by SEM. SEM images are given in Figure 2. It was observed that the obtained BN NSs were clearly spherical, while the NSs coated with chitosan were more layered with the effect of the coating. The SEM images were examined and it was determined that they were in full agreement with the size information in Table 3.

Another parameter that can affect the interaction of NSs with cells is the shape of the particles. First, changing the shape also affects the sizes of the nanomaterial. This means that volume, maximum diameter, or a combination of sizes must be held constant to compare the intake of differently shaped objects. Second, nonspherical objects can interact with the cell membrane in different directions. Thus, the contact area between the nano-sized object and the cell surface differs depending on the orientation when interacting with the cell membrane. In these cases, it is thought that different mechanisms are triggered depending on the orientation of the nanoparticles. Depending on the energy required for uptake from the membrane, it has been reported that the uptake will be highest in spherical-shaped nanoparticles, followed by cubic structures and then rod-shaped ones [39].

### 3.7. FTIR analysis of NS formulations

**Figure 3:**
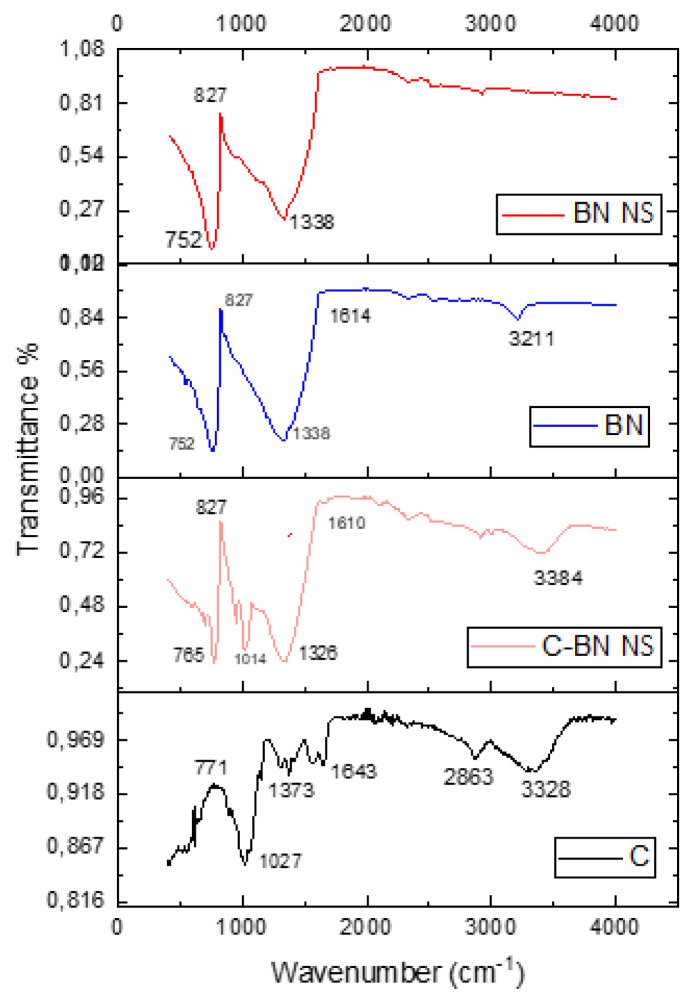
FTIR spectra of pure substances and formulations.

The pure BN, pure chitosan, and most stable NS formulations (N4 and N5) which were obtained as a result of stability studies were investigated by FTIR. FTIR spectrums are given in Figure 3. As expected in the FTIR spectrum of the lyophilized NS samples, the absorption of the light from the O-H bond was recorded at around 3300 cm^−1^. In addition, vibrations were observed of C-O and C-C bonds around 1000–1050 cm^−1^ and C-H bending vibrations around 1350–1400 cm^−1^ at pure chitosan and chitosan-coated BN NS. The characteristic amide band around ~1640 cm^−1^ is seen in both pure chitosan and chitosan-coated BN NSs [37]. In the BN and BN NS spectra, B-N-B and B-N bending vibrations and characteristic stretching were observed at ~ 750 cm^−1^ and ~1330 cm^−1^, respectively. The BN NS structure did not change at these voltages [10]. The FTIR spectra of the BN NSs did not show a significant shift in the position of the functional groups. The peaks indicated that there was no significant interaction between BN and surfactants and between BN and chitosan in the formulations. These data clearly show that chitosan-coated BN NSs have characteristic peak properties of both BN NSs and chitosan. It was observed that there was no undesirable interaction and the presence of chitosan confirmed the success of the coating. In conclusion, it has been shown that negatively charged BN NSs can be electrostatically bound with positively charged chitosan.

### 3.8. Evaluation of long-term stability of NS formulations

**Figure 4:**
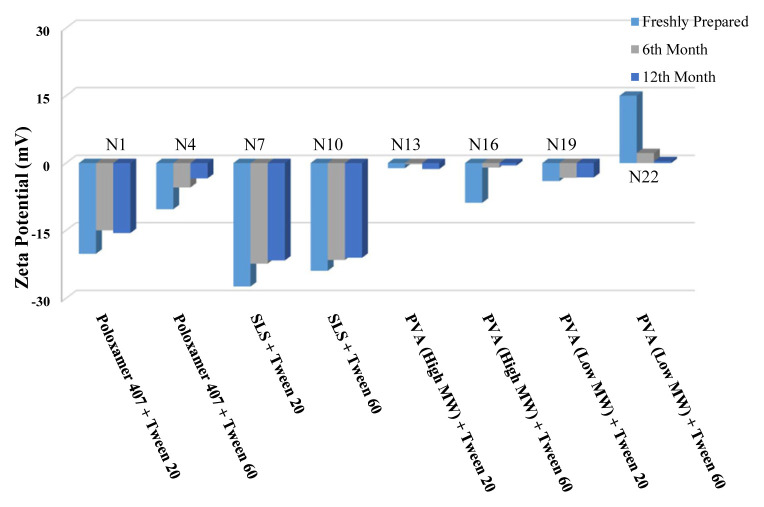
Effect of surfactants on zeta potential stability at +25 °C for BN NSs.

**Figure 5:**
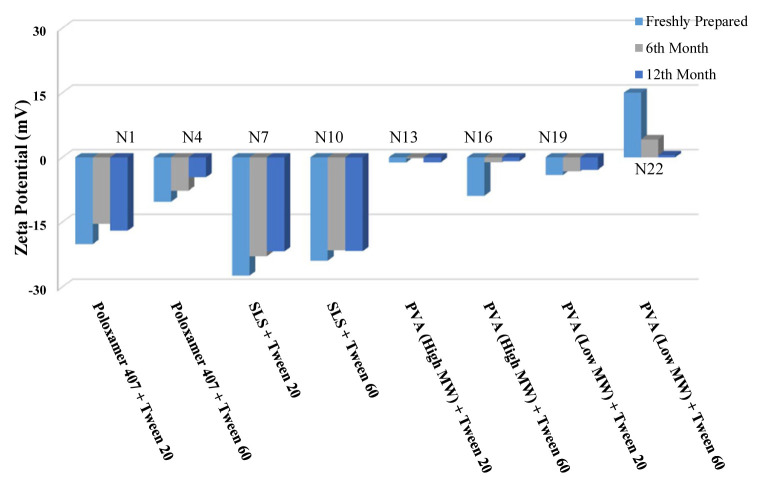
Effect of surfactants on zeta potential stability at +4 °C for BN NSs.

**Figure 6:**
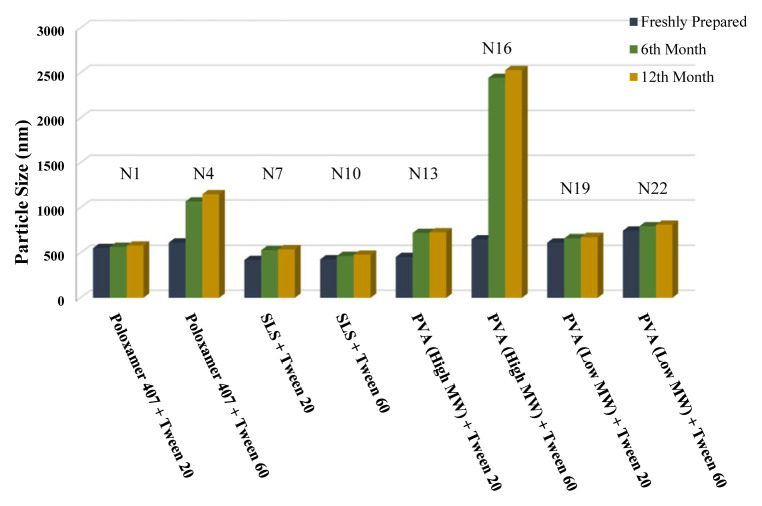
Effect of surfactants on particle size stability at +25 °C for BN NSs.

**Figure 7:**
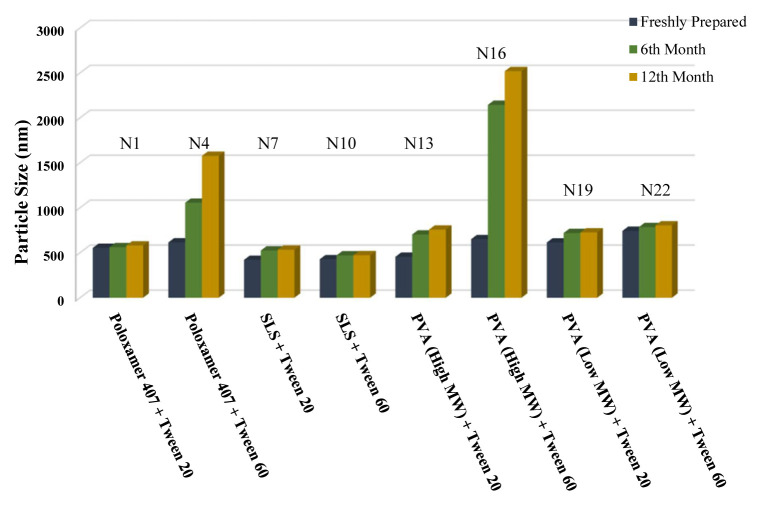
Effect of surfactants on particle size stability at +4 °C for BN NSs.

**Figure 8:**
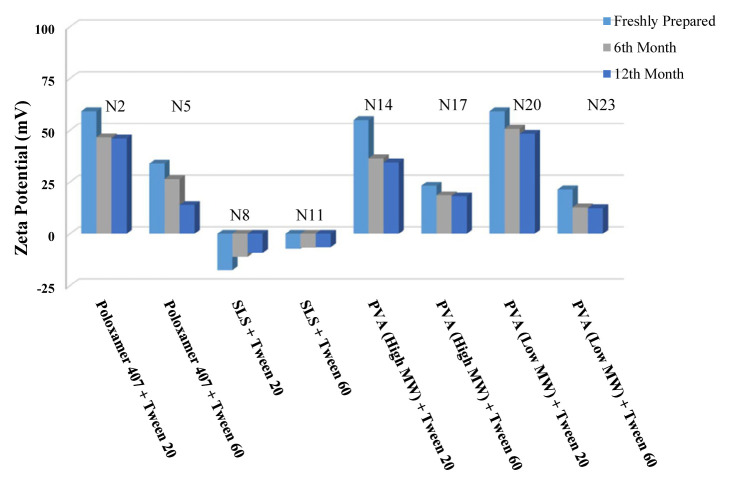
Effect of 10 mg chitosan coating on zeta potential stability at +25 °C for chitosan-coated BN NSs.

**Figure 9:**
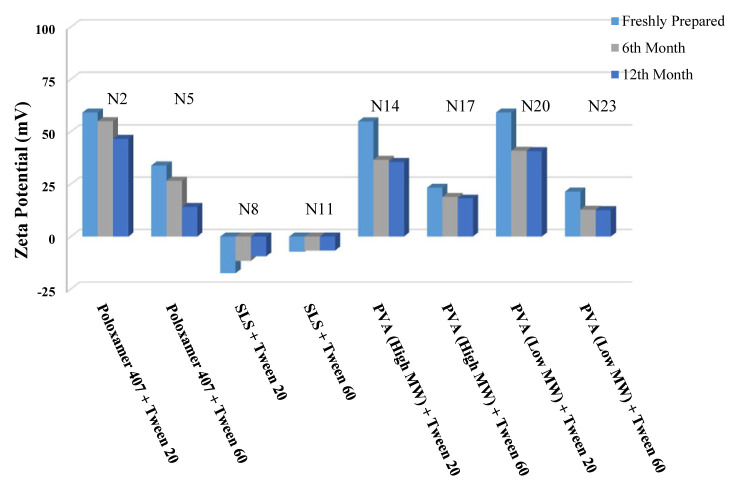
Effect of 10 mg chitosan coating on zeta potential stability at +4 °C for chitosan-coated BN NSs.

**Figure 10:**
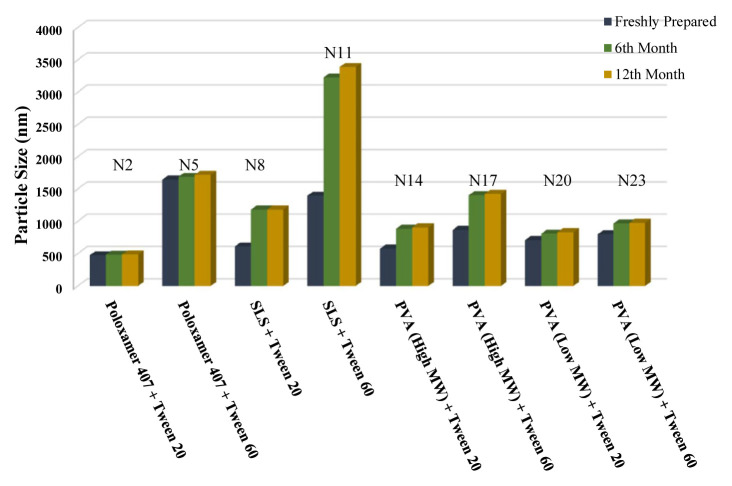
Effect of 10 mg chitosan coating on particle size stability at +25 °C for chitosan-coated BN NSs.

**Figure 11:**
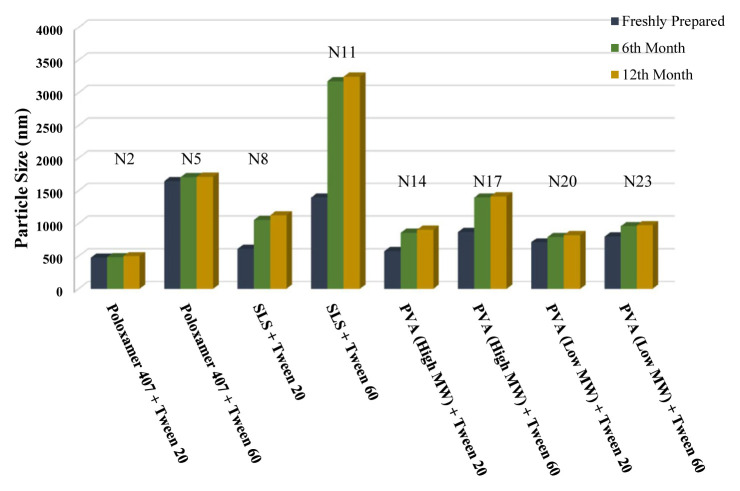
Effect of 10 mg chitosan coating on particle size stability at +4 °C for chitosan-coated BN NSs.

**Figure 12:**
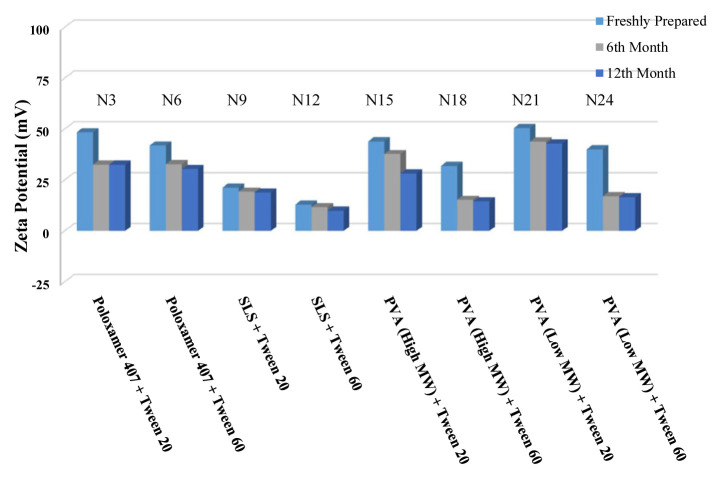
Effect of 20 mg chitosan coating on zeta potential stability at +25°C for chitosan-coated BN NSs.

**Figure 13:**
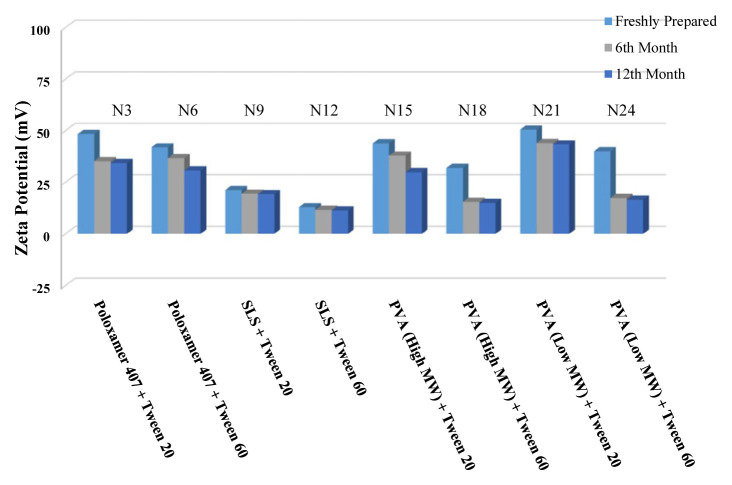
Effect of 20 mg chitosan coating on zeta potential stability at +4 °C for chitosan-coated BN NSs.

**Figure 14:**
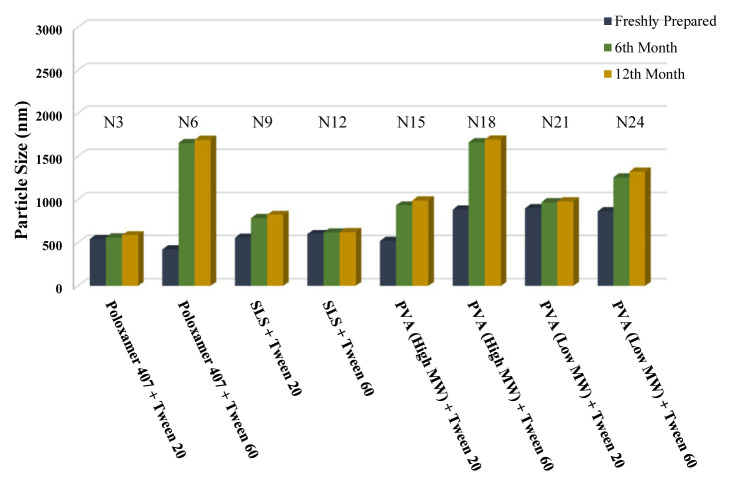
Effect of 20 mg chitosan coating on particle size stability at +25 °C for chitosan-coated BN NSs.

**Figure 15:**
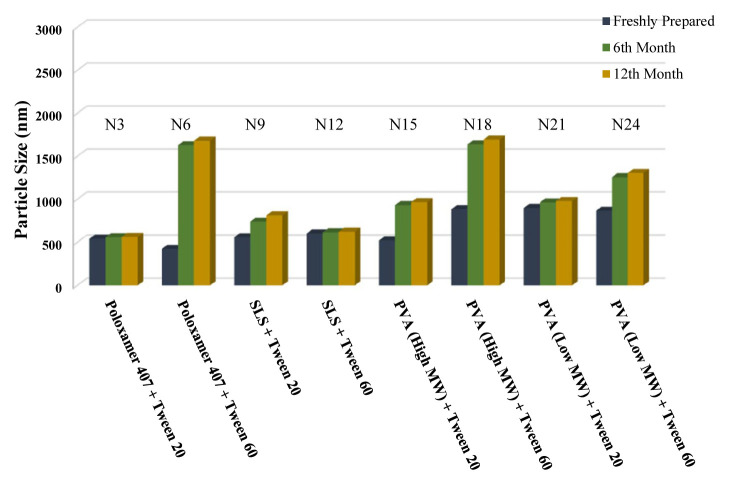
Effect of 20 mg chitosan coating on particle size stability at +4 °C for chitosan-coated BN NSs.

The long-term stability of the prepared NSs at both room temperature (25 °C) and +4 °C was evaluated and the results are given in Table 4, Table 5, and Table 6, along with the results of the freshly prepared samples. The graphs prepared based on the results have been graphed in order to better show the effects of surfactants and chitosan (Figures 4–15). Precipitation was observed under all conditions, but the formulations were easily reconstituted when reshaken. It has been reported that amorphous particles are more prone to aggregation [26].

Comprehensive and within specified restrictions for a product during shelf-life stability testing is required to confirm the suitability of drug ingredients up to the expiration date [3]. It has been reported in previous studies that the solid state is generally preferred over the aqueous nanosuspension formulation due to reduced aggregation of particles and other stability problems such as hydrolysis [57]. Stability studies were carried out in a nanosuspended state in our study due to examine the effect of stability and temperature in the aqueous medium on the particle size, PDI, and zeta potential of NSs over 6 and 12 months. In general, the size and zeta potentials of the formulations were found to be more stable at +4 °C than at +25 °C. Here, it is thought that NS mobility increases with increasing temperature, and the possibility of agglomeration is related to this situation [50].

Nanoparticular systems are thermodynamically unstable colloidal dispersions that are amenable to coalescence due to the Ostwald ripening phenomenon and positive Gibbs free energy exchange. The stabilizers used during preparation or storage should be able to adsorb on the surfaces of the NSs and provide steric or electrostatic stabilization. Generally, in addition to the steric barrier, strong and rapid adsorption of particles with stabilizers, and a long desorption time are required to achieve effective stabilization [51].

Steric stabilization can be achieved with nonionic surfactants and electrostatic repulsion with ionic surfactants. In this way, an increase in the stability of NSs can be observed. It has been noted in our formulations, that the stability is preserved significantly in terms of zeta potential and particle sizes with the combination of the action mechanisms of these stabilizers (such as N1–N12) [50]. However, an increase in size and a significant decrease in the zeta potential of chitosan-coated NSs were observed at +4 °C to +25 °C and for 6 to 12 months, especially at 20 mg chitosan-coated BN NSs. This may be caused by the lack of sufficient surfactant to keep the excess chitosan on the particle surface sterically and electrostatically stable. Because the changes in size and zeta potential of BN NSs coated with 10 mg chitosan were not as significant as those of 20 mg chitosan-coated BN NSs.

In previous studies, it has been reported that PVA is effective in the small size and stability of nanoparticles prepared with HPMC. A similar finding was reported that the particle size of nanoparticles prepared with Poloxamer 188 was larger than those prepared with PVA [50]. In our study, it was observed that Poloxamer 407, SLS, and PVA used during the preparation of BN NSs did not make a significant difference in size. In chitosan-coated BN NSs, it was determined that the formulations prepared with PVA in general were larger in size compared to the chitosan-coated formulations prepared with SLS or Poloxamer 407. However, considering the stability times, it was found that the particle size in formulations prepared with PVA (especially high MW PVA) increased significantly in both temperature conditions at 6 and 12 months. This may be due to the weakening of the steric barrier caused by the high molecular weight and long side chains of PVA, and the lack of sufficient other surfactants that offers electrostatic attraction force to protect the structure for a long time.

The dispersion and stability of nanoparticles are essential for their application as carriers in drug delivery systems. In our study, we observed that the chitosan coating significantly changed the distribution and stability of BN in an aqueous solution. Here, the interaction of amino groups in chitosan with the surface of BN NSs may play an important role. Depending on the type of surfactants used, the functional groups of chitosan adsorbed on the surface of BN NSs resulted in a good particle size distribution and a high zeta potential. Chitosan molecules can act here as polycationic surfactants and improve the stability of BN NSs [58]. In addition, chitosan adsorption triggers entropic repulsion between chitosan-coated BN NSs and stabilizes the particles [59]. In this way, the chitosan coating can enhance the ability of BN NSs to serve as a drug delivery system.

### 3.9. In vitro cytotoxicity assay

**Figure 16:**
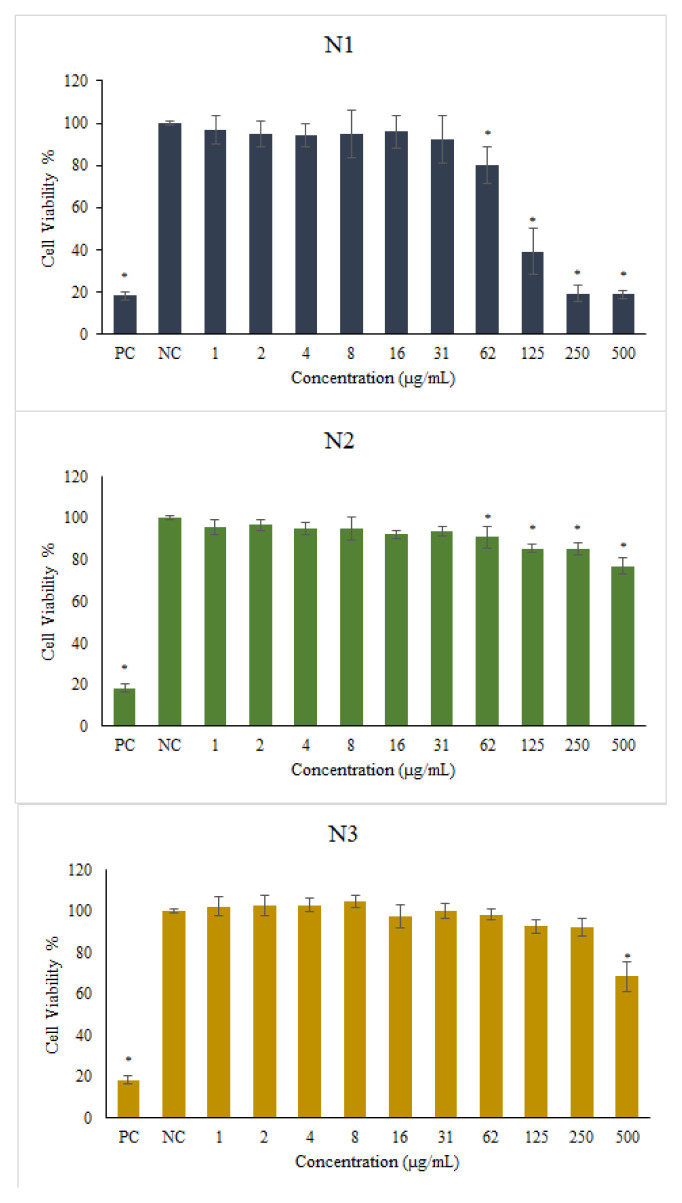
MTT test results of BN NS and chitosan-coated BN NS. (NC), negative control cells grown in only growth medium. (PC), positive control cells treated with 1% Triton™ X-100. Statistical significance was shown as *(p < 0.05) compared to the negative control.

The MTT assay is used to assess cell viability. Evaluation of cell viability was made by accepting the data obtained for the negative control group as 100% and comparing the other data by proportioning accordingly [60–62]. As a result of stability studies, the most stable formulations were selected and used (N1, N2, and N3) in cell culture studies. The obtained results are given in Figure 16. Only nanoparticles prepared with BN (N1) decreased cell viability at high doses (500 to 62 μg/mL). However, when the nanoparticles were coated with 10 mg chitosan, a significant improvement was observed in cell viability. In particular, it has been determined that BN nanoparticles, which were covered with 20 mg chitosan, maintain cell viability even at a dose of 250 μg/mL. This situation is also stated in the literature by Kim et al. They have coated BN nanoparticles using glycol chitosan and hyaluronic acid to improve BN’s stability and solubility problems. In this way, they have reported that cell viability has increased [63].

Similar cytotoxicity studies with BN-containing nanomaterials have been reported in the literature. As a result, it was observed that toxic effects started at similar doses in healthy and cancer cell lines. In a review of the studies on BN NS in the literature, it was reported that cell viability was preserved up to 90% even at a concentration of 100 μg/mL. Moreover, these BN NSs were injected into male rabbits and blood analyzes were performed for 3 days and detected no signs of toxicity or life dysfunction [7]. While Agustine et al. reported that the LC20 dose of BN nanotubes was 10 μg/mL, in our study the LC20 dose was found to be 62 μg/mL [64]. Ciofani et al. reported cell viability results on BN nanotubes, neuroblastoma cells, and human umbilical vessel epithelial cells. They reported that doses of 50 and 100 μg/mL were toxic for both cell types. They reported that concentrations up to 20 μg/mL did not have any adverse effects on cell metabolism or cell morphology after 24 h. They also reported that no change was observed in neuroblastoma cell differentiation [65]. In our study, cell viability was found above 80% even at higher doses (31 and 62 μg/mL). In this respect, the cell compatibility of BN NSs in our study was found to be better even at high doses compared to BN studies in the literature.

It is seen that the NSs (N1) prepared with only BN in Figure 4 significantly abolished cell viability in the first 4 concentrations compared to the negative control group. This has the potential to destroy cells, especially in cancerous tissues. On the contrary, in BN NSs coated with chitosan (N2), this situation achieved over 80% cell viability compared to the negative control group even at the first 4 concentrations. In the N3 formulation, cell viability due to the increase in the amount of chitosan did not make a significant difference compared to the negative control group, except for the first concentration. As can be understood from this, the presence of chitosan in the treatment with BN eliminates cytotoxicity. At the same time, it can create a prolonged effect in terms of showing the effect of BN for a longer period of time. Cell viability was preserved even at very high concentrations. With the coating of chitosan on BN NS, its uptake into the cell may also be facilitated. The obtained positive zeta potential has a significant advantage in entering the cell [42]. It is seen that not only the zeta potential but also chitosan, which is a natural polymer, contributes to this situation due to its nature. It was observed that cell viability was not toxic to cells at all concentrations starting from 250 μg/mL, especially when the same amount of chitosan was coated with the BN amount. In other words, no cytotoxic effect was observed at any dose except 500 μg/mL. The survival rate of cells is over 80%, even close to 100%. Ciofani et al. reported that a 50 μg/mL concentration of BN nanotubes coated with chitosan provides 80% cell viability, while a 100 μg/mL concentration provides 60% cell viability [66]. However, in our study, over 90% cell viability was detected even at 250 μg/mL BN concentration. It has been shown that BN NSs can be used safely in treatments in the medical field thanks to the chitosan coating without toxic effects caused by BN even at high doses.

Investigating the biological safety of drug delivery vehicles is critical for drug delivery systems. Although previous studies have shown that BN NS and chitosan are biocompatible and suitable for drug delivery, it is still necessary to investigate the safety of chitosan-coated BN NS because we used acetic acid to dissolve chitosan preparations in this study. Although high MW chitosan preparations are reported with potential cytotoxicity, BN NSs coated with chitosan of different molecular weights showed similar biocompatibility and no detectable cytotoxicity when added to cell cultures [10]. These in vitro results show that BN NS and chitosan-coated BN NSs are safe and suitable vehicles for drug delivery.

In the literature, there are studies on combining BN with chitosan. Zhang et al. have used BN nanoparticles as carriers on the cytokine-inducing ability of CpG oligodeoxynucleotides. To increase the interaction with cytokines, three different molecular weight chitosans were investigated [10]. Kisku et al. have designed a composite material by combining chitosan and BN via synthesis. They have reported the effects on oxygen permeability in food packaging [67]. However, long-term stability studies were not performed in any of these studies. Our study, unlike these studies, is also a formulation development study. The long-term effects of different surfactants were studied for stability. The effects of chitosan coating on BN NSs were also investigated. Increasing the amount of chitosan was investigated for stability with surfactants. At this stage, extreme changes in particle sizes have allowed formulation choices to be determined. Since the medicinal effects of BN and chitosan are known, cytotoxicity studies were conducted on the three most stable formulations. In the selected formulations in our study, it was observed that the particle size and zeta potential values increased in the positive direction, especially with the increase in the amount of chitosan (N2 and N3). The fact that BN NS (N1) decreases cell viability at high doses seems to change this situation. Due to the increased amount of chitosan, BN NSs continued to have cell viability even at high doses. Figure 16 shows that BN NSs and doses of 62, 125, and 250 μg/mL significantly affect cell viability (N1). However, it was determined that even these doses did not change the viability of the cells statistically significantly compared to the negative control with the N3 formulation. These data provided information on the long-term safe use of this formulation (at least 12 months) in all medical fields where the use of BN may be required in the future.

## 4. Conclusions

We report here in the study, that 24 different BN NS formulations, both chitosan-coated and non-chitosan-coated with 6 different surfactants, were successfully prepared with a high yield and characterized. The low cellular toxicity of BN NSs, especially chitosan-coated BN NSs, at higher concentrations shows that they have enormous potential in the diagnosis and treatment of diseases with boron-based compounds in the future. In addition, it is important that BN and chitosan have potential antimicrobial properties and that the prepared NS systems have both negative and high positive zeta potential. In particular, chitosan-coated BN NS have a high positive zeta potential, making them potential agents against Gram-positive and Gram-negative bacteria. Considering the surface charge densities, chitosan-coated BN NSs are likely to be effective against Gram-negative bacteria and BN NS against Gram-negative bacteria [68, 69].

The changes in zeta potential and particle sizes with the effect of surfactants and chitosan in nanosuspensions whose stability was evaluated were examined. In particular, prepared with Poloxamer 407 + Tween (N1 – N6) formulations were found to be the most stable formulations for 6 and 12 months, both at +4 °C and 25 °C.

In summary, in this study the effects of the selected surfactants and the used amount of chitosan during the preparation of formulations were investigated on the long-term stability of BN NSs and cell viability. Considering the results obtained from cytotoxicity and stability, further investigations of BN NSs and chitosan-coated BN NSs for use in medically preclinical and clinical studies should be evaluated.

## Figures and Tables

**Table 1:** BN containing NSs and their components (mg).

Formulation code	BN	Chitosan	Poloxamer 407	SLS	PVA (low MW)	PVA (high MW)	Tween 20	Tween 60
**N1**	20	-	15	-	-	-	35	-
**N2**	20	10	15	-	-	-	35	-
**N3**	20	20	15	-	-	-	35	-
**N4**	20	-	15	-	-	-	-	35
**N5**	20	10	15	-	-	-	-	35
**N6**	20	20	15	-	-	-	-	35
**N7**	20	-	-	15	-	-	-	35
**N8**	20	10	-	15	-	-	-	35
**N9**	20	20	-	15	-	-	-	35
**N10**	20	-	-	15	-	-	35	-
**N11**	20	10	-	15	-	-	35	-
**N12**	20	20	-	15	-	-	35	-
**N13**	20	-	-	-	-	15	35	-
**N14**	20	10	-	-	-	15	35	-
**N15**	20	20	-	-	-	15	35	-
**N16**	20	-	-	-	-	15	-	35
**N17**	20	10	-	-	-	15	-	35
**N18**	20	20	-	-	-	15	-	35
**N19**	20	-	-	-	15	-	35	-
**N20**	20	10	-	-	15	-	35	-
**N21**	20	20	-	-	15	-	35	-
**N22**	20	-	-	-	15	-	-	35
**N23**	20	10	-	-	15	-	-	35
**N24**	20	20	-	-	15	-	-	35

**Table 2:** Contents and quantities of formulations used for cell culture.

Code	Formulation	Contents	Concentration	Storage condition
**N1**	BN NS	BN	4 mg/mL	+4 °C
**N2**	Chitosan-coated BN NS	BN + Chitosan	4 + 2 mg/mL	+4 °C
**N3**	Chitosan-coated BN NS	BN + Chitosan	4 + 4 mg/mL	+4 °C

**Table 3:** Yields, zeta potential, PDI, and particle size analysis results of BN NS and chitosan-coated BN NS formulations (n = 3, +25 °C, Ⴟ±SD).

Formulation code	Yield (%)	Zeta potential (mV)	PDI	Particle size (nm)
**N1**	91.88 ± 1.91	−20.1 ± 0.68	0.268 ± 0.034	552.2 ± 2.02
**N2**	87.69 ± 2.48	59.2 ± 0.99	0.251 ± 0.007	473.6 ± 1.50
**N3**	66.41 ± 3.15	48.4 ± 0.61	0.290 ± 0.035	540.9 ± 3.12
**N4**	93.15 ± 0.89	−10.3 ± 0.32	0.116 ± 0.032	613.7 ± 3.85
**N5**	89.72 ± 1.85	33.9 ± 3.18	0.293 ± 0.039	1645.0 ± 12.77
**N6**	65.66 ± 2.87	41.9 ± 0.72	0.414 ± 0.020	422.0 ± 11.96
**N7**	94.21 ± 1.15	−27.4 ± 1.65	0.198 ± 0.036	420.4 ± 5.73
**N8**	86.49 ± 0.89	−17.5 ± 0.36	0.625 ± 0.080	610.4 ± 19.62
**N9**	61.83 ± 1.93	21.2 ± 0.21	0.175 ± 0.013	556.6 ± 6.31
**N10**	88.57 ± 2.15	−23.9 ± 0.40	0.218 ± 0.008	426.7 ± 2.214
**N11**	84.08 ± 2.74	−7.19 ± 0.13	0.268 ± 0.018	1396 ± 33.72
**N12**	60.39 ± 1.88	12.9 ± 0.40	0.242 ± 0.028	600.4 ± 4.76
**N13**	94.11 ± 0.19	−1.15 ± 0.12	0.188 ± 0.030	454.1 ± 5.96
**N14**	87.48 ± 1.61	54.9 ± 1.54	0.221 ± 0.012	576.1 ± 9.37
**N15**	68.97 ± 1.98	43.9 ± 0.92	0.213 ± 0.004	521.6 ± 16.67
**N16**	90.76 ± 0.57	−8.87 ± 0.52	0.217 ± 0.011	649.6 ± 9.60
**N17**	82.19 ± 2.35	23.2 ± 0.21	0.198 ± 0.031	865.8 ± 5.40
**N18**	65.91 ± 2.18	31.9 ± 0.25	0.244 ± 0.009	883.3 ± 48.01
**N19**	91.45 ± 2.61	−4.03 ± 0.62	0.198 ± 0.015	612.6 ± 2.35
**N20**	86.79 ± 2.87	59.2 ± 0.31	0.277 ± 0.080	709.4 ± 38.88
**N21**	71.56 ± 3.15	50.5 ± 1.15	0.225 ± 0.042	898.1 ± 29.09
**N22**	93.78 ± 2.09	15.0 ± 0.32	0.333 ± 0.059	741.6 ± 18.05
**N23**	89.04 ± 2.47	21.4 ± 1.40	0.277 ± 0.015	799.4 ± 21.73
**N24**	69.41 ± 3.67	40.0 ± 0.61	0.186 ± 0.029	865.4 ± 4.41

**Table 4:** Zeta potential stability of BN NS and chitosan-coated BN NS formulations (mV, Ⴟ±SD).

Formulation code	Freshly prepared	6th month		12th month	
		+25 °C	+4 °C	+25 °C	+4 °C
**N1**	−20.1 ± 0.68	−15.0 ± 0.05	−15.4 ± 0.08	−15.6 ± 0.15	−17.0 ± 0.11
**N2**	59.2 ± 0.99	46.6 ± 1.21	55.0 ± 1.10	46.0 ± 2.23	46.6 ± 1.87
**N3**	48.4 ± 0.61	32.5 ± 3.71	35.2 ± 2.58	32.5 ± 2.95	34.3 ± 1.88
**N4**	−10.3 ± 0.32	−5.45 ± 0.39	−7.73 ± 0.12	−3.42 ± 1.15	−4.60 ± 0.98
**N5**	33.9 ± 3.18	26.4 ± 4.48	26.6 ± 3.66	13.9 ± 2.86	14.1 ± 2.13
**N6**	41.9 ± 0.72	32.7 ± 1.18	36.6 ± 1.01	30.4 ± 1.27	30.7 ± 0.99
**N7**	−27.4 ± 1.65	−22.3 ± 0.88	−22.9 ± 1.05	−21.6 ± 1.16	−21.8 ± 1.29
**N8**	−17.5 ± 0.36	−11.1 ± 0.75	−11.6 ± 0.59	−9.2 ± 1.42	−9.5 ± 1.23
**N9**	21.2 ± 0.21	19.2 ± 0.46	19.4 ± 0.24	18.8 ± 0.87	19.2 ± 0.67
**N10**	−23.9 ± 0.40	−21.5 ± 0.49	−21.5 ± 0.19	−21.0 ± 0.27	−21.7 ± 0.14
**N11**	−7.19 ± 0.13	−6.67 ± 0.78	− 6.69 ± 0.59	−6.5 ± 0.41	−6.67 ± 0.64
**N12**	12.9 ± 0.40	11.6 ± 0.17	11.6 ± 0.48	9.88 ± 0.93	11.3 ± 0.15
**N13**	−1.15 ± 0.12	−0.233 ± 0.08	−0.224 ± 0.10	−1.34 ± 0.11	1.11 ± 0.19
**N14**	54.9 ± 1.54	36.4 ± 2.38	36.5 ± 3.21	34.5 ± 2.11	35.5 ± 2.98
**N15**	43.9 ± 0.92	37.7 ± 1.11	37.9 ± 1.23	28.2 ± 0.89	29.9 ± 1.01
**N16**	−8.87 ± 0.52	−1.01 ± 0.87	−1.10 ± 0.65	−0.541 ± 0.48	−0.876 ± 0.61
**N17**	23.2 ± 0.21	18.6 ± 0.39	18.9 ± 0.42	18.1 ± 0.88	18.1 ± 0.71
**N18**	31.9 ± 0.25	15.2 ± 1.23	15.5 ± 1.09	14.5 ± 2.71	15.0 ± 2.20
**N19**	−4.03 ± 0.62	−3.29 ± 0.53	−3.21 ± 0.27	−2.63 ± 0.09	− 2.89 ± 0.12
**N20**	59.2 ± 0.31	50.7 ± 1.13	52.9 ± 0.81	48.3 ± 0.99	49.7 ± 0.76
**N21**	50.5 ± 1.15	43.8 ± 3.11	44.0 ± 2.28	42.9 ± 2.45	43.3 ± 1.69
**N22**	15.0 ± 0.32	2.22 ± 0.55	4.17 ± 0.23	0.406 ± 0.13	0.516 ± 0.11
**N23**	21.4 ± 1.40	12.7 ± 0.88	12.8 ± 0.71	12.3 ± 1.12	12.6 ± 1.01
**N24**	40.0 ± 0.61	17.0 ± 0.94	17.3 ± 1.17	16.5 ± 1.52	16.5 ± 1.09

**Table 5:** PDI stability of BN NS and chitosan-coated BN NS formulations (Ⴟ±SD).

Formulation code	Freshly prepared	6th Month		12th Month	
		+25 °C	+4 °C	+25 °C	+4 °C
**N1**	0.268 ± 0.034	0.329 ± 0.041	0.263 ± 0.037	0.249 ± 0.019	0.261 ± 0.014
**N2**	0.251 ± 0.007	0.250 ± 0.018	0.245 ± 0.020	0.255 ± 0.059	0.254 ± 0.028
**N3**	0.290 ± 0.035	0.260 ± 0.029	0.254 ± 0.015	0.248 ± 0.016	0.273 ± 0.041
**N4**	0.116 ± 0.032	0.290 ± 0.070	0.289 ± 0.041	0.252 ± 0.028	0.256 ± 0.039
**N5**	0.293 ± 0.039	0.277 ± 0.031	0.277 ± 0.027	0.284 ± 0.019	0.318 ± 0.045
**N6**	0.414 ± 0.020	0.191 ± 0.081	0.230 ± 0.075	0.257 ± 0.052	0.278 ± 0.039
**N7**	0.198 ± 0.036	0.264 ± 0.035	0.283 ± 0.042	0.264 ± 0.021	0.268 ± 0.029
**N8**	0.625 ± 0.080	0.174 ± 0.042	0.196 ± 0.051	0.174 ± 0.062	0.196 ± 0.069
**N9**	0.175 ± 0.013	0.252 ± 0.046	0.167 ± 0.019	0.256 ± 0.031	0.233 ± 0.052
**N10**	0.218 ± 0.008	0.193 ± 0.012	0.216 ± 0.016	0.192 ± 0.021	0.217 ± 0.040
**N11**	0.268 ± 0.018	0.283 ± 0.029	0.330 ± 0.051	0.287 ± 0.019	0.303 ± 0.033
**N12**	0.242 ± 0.028	0.234 ± 0.011	0.214 ± 0.041	0.241 ± 0.009	0.187 ± 0.027
**N13**	0.188 ± 0.030	0.250 ± 0.048	0.316 ± 0.061	0.208 ± 0.023	0.280 ± 0.029
**N14**	0.221 ± 0.012	0.216 ± 0.008	0.173 ± 0.036	0.162 ± 0.041	0.237 ± 0.017
**N15**	0.213 ± 0.004	0.223 ± 0.010	0.229 ± 0.021	0.147 ± 0.031	0.191 ± 0.009
**N16**	0.217 ± 0.011	0.457 ± 0.061	0.465 ± 0.069	0.532 ± 0.088	0.500 ± 0.071
**N17**	0.198 ± 0.031	0.313 ± 0.019	0.298 ± 0.009	0.298 ± 0.012	0.360 ± 0.025
**N18**	0.244 ± 0.009	0.304 ± 0.029	0.442 ± 0.019	0.323 ± 0.012	0.303 ± 0.041
**N19**	0.198 ± 0.015	0.147 ± 0.036	0.187 ± 0.029	0.193 ± 0.042	0.191 ± 0.033
**N20**	0.277 ± 0.080	0.261 ± 0.010	0.230 ± 0.023	0.405 ± 0.042	0.279 ± 0.013
**N21**	0.225 ± 0.042	0.282 ± 0.015	0.226 ± 0.028	0.235 ± 0.023	0.251 ± 0.011
**N22**	0.333 ± 0.059	0.230 ± 0.021	0.223 ± 0.011	0.261 ± 0.038	0.328 ± 0.052
**N23**	0.277 ± 0.015	0.320 ± 0.019	0.368 ± 0.056	0.321 ± 0.034	0.266 ± 0.008
**N24**	0.186 ± 0.029	0.325 ± 0.022	0.240 ± 0.019	0.247 ± 0.027	0.252 ± 0.019

**Table 6:** Particle size stability of BN NS and chitosan-coated BN NS formulations (nm, Ⴟ±SD).

Formulation code	Freshly prepared	6th Month		12th Month	
		+25 °C	+4 °C	+25 °C	+4 °C
**N1**	552.2 ± 2.02	566.1 ± 3.77	560.9 ± 2.89	580.2 ± 4.45	579.6 ± 3.71
**N2**	473.6 ± 1.50	483.4 ± 2.22	482.7 ± 2.38	486.9 ± 1.12	498.0 ± 1.98
**N3**	540.9 ± 3.12	559.5 ± 3.01	557.1 ± 2.95	587.5 ± 1.99	560.7 ± 2.15
**N4**	613.7 ± 3.85	1068.0 ± 11.50	1055.0 ± 12.98	1148.0 ± 9.81	1577.0 ± 23.12
**N5**	1645.0 ± 12.77	1687.0 ± 14.11	1705.0 ± 10.97	1719.0 ± 11.19	1712.0 ± 15.72
**N6**	422.0 ± 11.96	1657.0 ± 22.45	1627.0 ± 19.15	1694.0 ± 18.41	1680.0 ± 15.78
**N7**	420.4 ± 5.73	529.7 ± 4.93	524.2 ± 5.61	538.3 ± 3.88	532.9 ± 6.12
**N8**	610.4 ± 19.62	1181.0 ± 8.54	1052.0 ± 13.45	1181.0 ± 12.89	1122.0 ± 10.85
**N9**	556.6 ± 6.31	784.2 ± 5.15	738.8 ± 7.11	823.5 ± 4.13	812.0 ± 6.62
**N10**	426.7 ± 2.21	465.5 ± 3.16	469.5 ± 2.78	478.9 ± 3.11	471.3 ± 1.95
**N11**	1396.0 ± 33.72	3228.0 ± 54.16	3175.0 ± 48.65	3390.0 ± 59.81	3244.0 ± 47.12
**N12**	600.4 ± 4.76	618.3 ± 3.25	613.7 ± 2.75	623.1 ± 1.78	620.6 ± 2.12
**N13**	454.1 ± 5.96	716.7 ± 3.32	701.7 ± 4.95	723.9 ± 7.75	756.1 ± 4.31
**N14**	576.1 ± 9.37	883.5 ± 11.41	855.5 ± 13.12	904.1 ± 18.41	900.9 ± 15.66
**N15**	521.6 ± 16.67	932.9 ± 9.72	931.0 ± 7.65	989.9 ± 11.44	965.1 ± 10.05
**N16**	649.6 ± 9.60	2442.0 ± 31.28	2144.0 ± 24.23	2529.0 ± 31.60	2519.0 ± 25.69
**N17**	865.8 ± 5.40	1404.0 ± 9.12	1396.0 ± 11.02	1425.0 ± 8.87	1414.0 ± 13.12
**N18**	883.3 ± 48.01	1666.0 ± 38.48	1637.0 ± 33.12	1696.0 ± 29.65	1693.0 ± 25.11
**N19**	612.6 ± 2.35	659.8 ± 6.69	647.6 ± 7.12	672.8 ± 5.91	665.8 ± 3.88
**N20**	709.4 ± 38.88	807.8 ± 41.64	791.3 ± 36.15	831.7 ± 45.28	820.2 ± 39.15
**N21**	898.1 ± 29.09	968.6 ± 22.20	961.9 ± 29.84	979.6 ± 15.91	977.9 ± 11.87
**N22**	741.6 ± 18.05	791.3 ± 8.85	783.9 ± 10.90	807.8 ± 11.38	802.8 ± 13.13
**N23**	799.4 ± 21.73	966.2 ± 18.15	956.5 ± 13.85	976.6 ± 15.98	970.9 ± 13.52
**N24**	865.4 ± 4.41	1257.0 ± 28.15	1255.0 ± 22.41	1325.0 ± 36.31	1305.0 ± 30.15
